# The ENDOMIX project: an interdisciplinary approach to understanding how real-life chemical mixtures target the immune system to trigger disease

**DOI:** 10.12688/openreseurope.19088.1

**Published:** 2024-12-19

**Authors:** Ana Claudia Zenclussen, Valentina Belmar Erilkin, Linda Böhmert, Petra Borilova Linhartova, Albert Braeuning, Georg Braun, Cécile Chevrier, Liesbeth Duijts, Beate Isabella Escher, Janine Felix, Sergio Gómez-Olarte, Mònica Guxens, Gunda Herberth, Klara Hilscherova, Jana Klanova, Yvonne Kohl, Katharina Krischak, Dominique Lagadic-Gossmann, Sophie Langouët, Sabrina Llop, Maria Jose Lopez-Espinosa, Léa Maitre, Corinne Martin-Chouly, Nicole Meyer, Marion Ouidir, Thi Anh Mai Pham, Claire Philippat, Raymond Pieters, Marie-Laure Pinel-Marie, Normand Podechard, Tobias Polte, Elliott Price, Oliver Robinson, Kristin Schubert, Anne Schumacher, Violeta Stojanovska, Tamara Tal, Paolo Vineis, Robert van Vorstenbosch, Roel Vermeulen, Charline Warembourg

**Affiliations:** 1 Department of Environmental Immunology, Helmholtz-Centre for Environmental Research - UFZ, Leipzig, Saxony, 04318, Germany; 2 German Center for Child and Adolescent Health (DZKJ), partner site Leipzig/Dresden, Leipzig, Germany; 3 European Institute for Biomedical Imaging Research (EIBIR), Vienna, Austria; 4 German Federal Institute for Risk Assessment (BfR), Dept. Food Safety, Berlin, 10589, Germany; 5 RECETOX, Faculty of Science, Masaryk University, Brno, South Moravian Region, Czech Republic; 6 Department of Cell Toxicology, Helmholtz-Centre for Environmental Research - UFZ, Leipzig, Saxony, 04318, Germany; 7 Univ Rennes, Inserm, EHESP, Institut de Recherche en Santé, Environnement et Travail - UMR_S 1085, Rennes, France; 8 Department of Pediatrics, Erasmus MC, University Medical Center, Rotterdam, The Netherlands; 9 Generation R Study Group, Erasmus MC, University Medical Center Rotterdam, Rotterdam, The Netherlands; 10 Department of Neonatal and Intensive Care, Division of Neonatology, Erasmus MC, University Medical Center, Rotterdam, Rotterdam, The Netherlands; 11 Institute for Global Health, ISGlobal, Barcelona, Spain; 12 Fraunhofer Institute for Biomedical Engineering IBMT, Sulzbach, Germany; 13 Epidemiology and Environmental Health Joint Research Unit, FISABIO–Universitat Jaume I–Universitat de València, Valencia, Spain; 14 Spanish Consortium for Research on Epidemiology and Public Health (CIBERESP), Madrid, Spain; 15 Department of Nursing, Faculty of Nursing and Chiropody, University of Valencia, Valencia, Spain; 16 University Grenoble Alpes, Inserm U-1209, CNRS-UMR-5309, Environmental Epidemiology Applied to Development and Respiratory Health Team, Institute for Advanced Biosciences, Grenoble, France; 17 Institute for Risk Assessment Sciences, Utrecht University, Utrecht, The Netherlands; 18 MRC Centre for Environment and Health, School of Public Health, Imperial College London, London, UK; 19 Department of Molecular Toxicology, Helmholtz-Centre for Environmental Research - UFZ, Leipzig, Saxony, 04318, Germany; 20 Department of Ecotoxicology, Helmholtz-Centre for Environmental Research - UFZ, Leipzig, Saxony, 04318, Germany

**Keywords:** endocrine disrupting chemicals, EDC exposure, chemical mixtures, immune system, long-term health effects, interdisciplinary approach, science to policy translation

## Abstract

The true impact of endocrine disrupting chemicals (EDCs) on human health is far from being understood. Humans are exposed to mixtures of chemicals throughout their lives, yet regulations and most studies focus on individual chemicals. ENDOMIX takes a novel approach to identifying associations and causality between EDCs and adverse health outcomes by focusing on exposure to mixtures of EDCs over the life course, including windows of susceptibility, using human biomonitoring data from several European cohorts. We will model and measure how real-life EDC mixtures act together and target the immune system to initiate, trigger or maintain disease. Health effects will be investigated using pioneering methodologies ranging from high-throughput *in vitro* bioassays, sophisticated organoid and co-culture systems, to *in vivo* models. In combination, they will provide valuable information on mechanistic pathways and transgenerational effects of EDC exposure. We aim to identify biomarkers and patterns of chemical exposures that are easy to measure, available for large cohorts and indicative for adverse health outcomes. We will use *in vitro*, *in silico* and *in vivo* data to strengthen causal inference using a weight-of-evidence approach. Moreover, using novel text mining methods, we will create knowledge graphs to capture and summarize the complexity of biomechanistic information, which aids rapid risk assessments and the creation of network models. The knowledge generated by ENDOMIX will provide an evidence base for policy-making and also reach people of all ages to raise awareness of the risks of EDC exposure and encourage health-promoting behaviors.

## Plain language summary

Endocrine disrupting chemicals (EDCs) are natural or man-made substances that mess with the normal function of the body’s hormone system in both humans and animals. The true effect of EDCs on human health is far from being understood. People are exposed to a mixture of many different chemicals over their lifetime, yet regulations and most research studies often look at one chemical at a time. The ENDOMIX project uses a new approach to study the link between a variety of EDCs and harmful effects on health throughout the person’s lifetime. We will use data from human biological monitoring of several European study groups. We will further study how real-life mixtures of EDCs act together on the immune system to cause or perpetuate diseases. We will generate and use data from laboratory experiments (in vitro, in silico)), computational experiments and animal experiments (in vivo) to better understand the cause-and-effect relationship. The evidence produced by ENDOMIX will be a solid foundation for policy-making and help to inform people of all ages about the risk of exposure to EDCs and encourage health-promoting behavior.

## Introduction

Endocrine disrupting chemicals (EDCs) are natural or synthetic chemicals that interfere with the normal functioning of the endocrine system in humans and animals. However, the impact of EDCs in general and man-made chemicals in particular on human health is not so well-understood. They are ubiquitous and have been implicated in the increase in the prevalence of common non-communicable diseases (NCDs) over the last decade ^1^. Man-made EDC chemicals, from here on referred to as EDCs, can be found not only in the environment (water, soil, air), but also in consumer products (plastics, food packaging, electronics and food packaging), in personal care products (e.g., sunscreen, cosmetics), as well as in food and drinking water ^2^. Efforts to minimize exposure to harmful EDCs include regulations on their use in consumer products, bans on certain chemicals, and public education campaigns to raise awareness of their potential adverse health risks. Many of these approaches rely on results obtained from scenarios on exposure to single chemicals or at best to a family of chemicals. In reality, however, we are exposed to multiple EDCs simultaneously throughout our lives. Not only do some EDCs accumulate in the environment and enrich in certain tissues ^3^, but their mixtures will lead to additive, and in rare cases, synergistic or antagonistic effects ^4^.

Deviations from normal healthy state or development do cause individual suffering and impose enormous costs on health care systems ^5^. Although ample evidence of the adverse effects of EDC exposure in wildlife and animals was already reported by the International Program on Chemical Safety in 2002 and has been complemented by a large number of epidemiological studies over the past decades, significant gaps still remain in our understanding of the health effects and mechanisms of EDC in the human population ^3, 6– 8^ The so-called immunotoxicology lists among the less explored topics. Indeed, the effects of EDCs on the immune system have been poorly investigated. However, dysregulated immune pathways may underlie and are important drivers of common human diseases. The fact that EDCs may affect the immune system and cause abnormalities relevant to disease is poorly addressed ^5^. Despite growing evidence feeding to his emergent field of research, more research is needed to fully understand the breadth of EDCs´ impact on the immune system, especially concerning long-term health effects and the combined impact of multiple disruptors to which we are exposed chronically. Additionally, understanding individual and group susceptibility needs to be considered.

In this article we present the concept and objectives of the ENDOMIX project to address the above-mentioned challenges and needs.

## ENDOMIX conception

The vision and aim of ENDOMIX is to thoroughly clarify and understand the overall immunotoxic or immunomodulatory impact of EDC mixtures and underlying mechanisms leading to adverse health outcomes. ENDOMIX is a cutting-edge research initiative that aims to uncover the true impact of EDCs on human health by bridging existing knowledge gaps between science and policy. It was born out of the need to synthesize available information from different approaches and sources, and the urgent demands of society to protect individual health by reducing exposure to and minimizing chemical risks. The project takes an interdisciplinary approach, covering the full chain from population-based studies to mechanistic understanding of EDC effects at the cellular and target organ level, and translating this into policy recommendations.

ENDOMIX strongly focuses on EDCs and their possible combined effects with other stressors, including socioeconomic aspects, and addresses an area that is under-researched and where knowledge is grossly lacking. Exposure to EDCs is associated with important adverse health outcomes in humans across the life course, including impaired respiratory and cardiometabolic health, neurodevelopment autoimmunity, and altered reproductive health ^9^. These constitute health outcomes with a large burden of disease worldwide ^10^. The exact underlying biological pathways are mostly unclear. Many studies overlook the fact that there are age windows in which exposure to EDCs are more critical, making certain populations like pregnant women, neonates, infants and children more vulnerable to their effects. ENDOMIX takes a unique approach to this problem by focusing on a) chemical mixtures (real life EDCs), b) immunotoxicity as a critical central mechanism, c) strong study designs, of multiple European cohorts with data on chemical exposures and NCDs across the life course, d) modelling approaches complemented by bioassays that provide novel knowledge about real-life mixtures and their effects, e) mechanistic and causal molecular investigation including *in vitro* barrier and target organ models as well as different animal species considering the 3R rules, and f) knowledge synthesis through novel text mining approaches for biomechanistic insights, modelling and risk estimation.

Existing gaps in scientific understanding, policy, and knowledge transfer hamper the effectiveness of European regulations in assessing, preventing, and reducing human and environmental exposure to EDCs. Addressing this uncertainty by integrating robust data sources, existing data from biobank samples, modelling strategies, and bioassays will be **transformative in identifying real-life mixtures that are of concern and should be looked at more carefully.** The effects of chemical mixtures may be more significant than their components, potentially causing irreversible adverse effects during vulnerable periods of life such as pregnancy, early childhood, and adolescence. This aligns with the Developmental Origins of Health and Disease (DOHaD) framework, which posits that many adult diseases originate in early life, with chemical exposure during these vulnerable periods potentially driving adverse health outcomes ^7, 11^. Adolescence is a key period of rapid and unique development, in which individuals may be very prone to major endocrine and metabolic changes, and thus potentially highly sensitive to EDCs, but this has rarely been examined in epidemiological studies. Further, it is not considered in the design of single *in vivo* studies nor experimental settings for risk assessment. ENDOMIX will therefore break new ground by focussing on puberty endpoints.

A recent report of the Lancet Commission for Planetary Health identified **immunotoxicity**, the focus of **ENDOMIX**, as one of “three particularly worrisome, and inadequately charted consequences of chemical pollution” ^12^, the other two being neurotoxicity and reproductive toxicity. Even though that it is documented that acute or chronic EDC exposure on the immune system results in autoimmune diseases ^13^, or common diseases like diabetes, obesity, allergies and respiratory diseases, the impact of EDCs on health outcomes mediated by the immune system is poorly acknowledged or completely overlooked in most of current approaches. ENDOMIX will advance the field by studying immunotoxicity as a driver of deviations in human health development following exposure to EDCs, and a perpetuator of pathologies.

**Figure 1.**
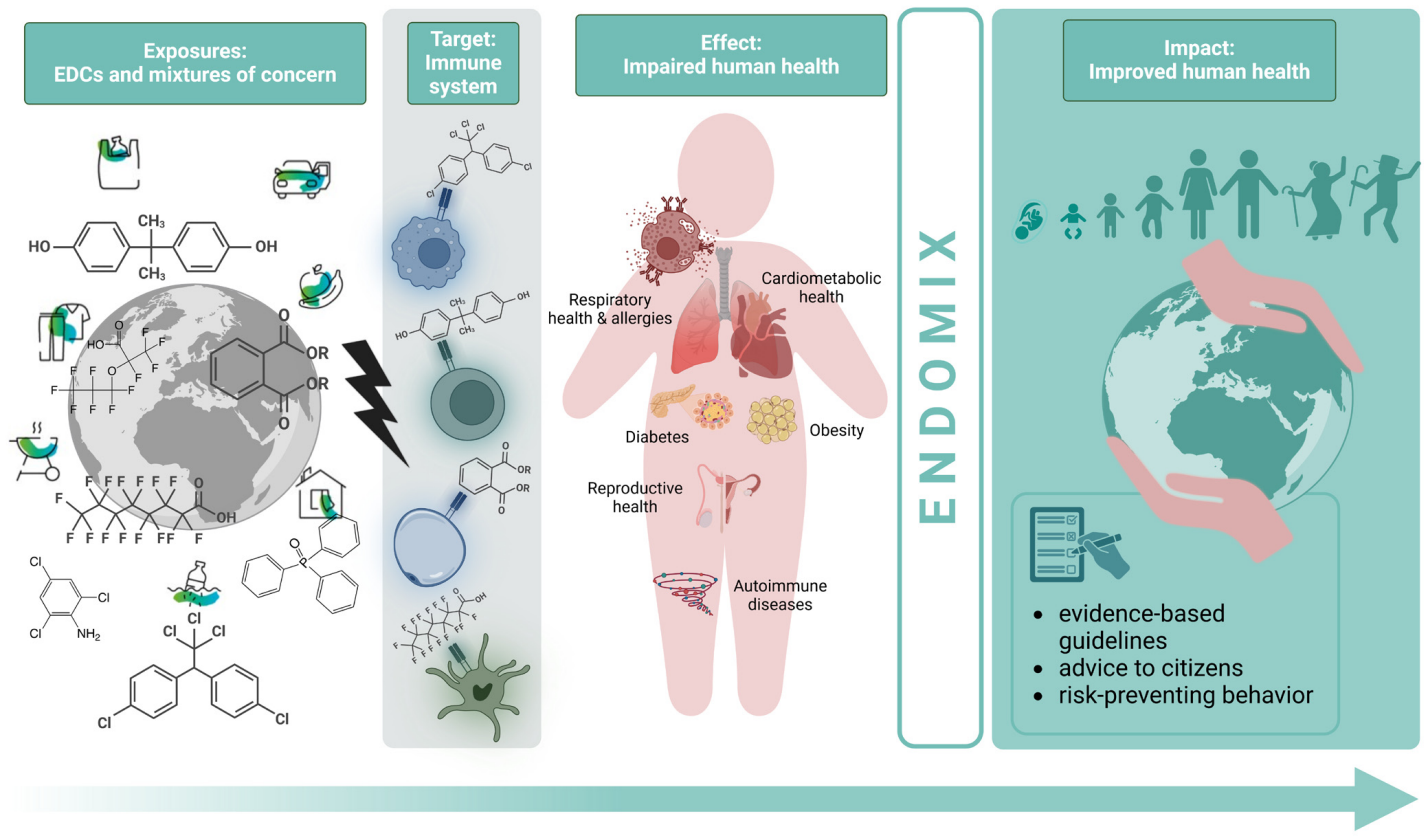
The ENDOMIX strategy from data generation to its translation into policy guidelines and recommendations. With a combination of life-course population-based cohort data, high-throughput screening and modelling to identify new mixtures of concern, *in vitro* cell, barrier and organoid models, *in vivo* models, omics, and computational approaches, ENDOMIX will deliver evidence-based guidelines and advice to citizens on improving human health.

Dysregulated immune pathways are known drivers of common diseases such as diabetes, obesity and respiratory diseases. In particular, dysregulated immune responses in pregnancy may have long-term effects on offspring, even if the pregnancy seemed normal. The fact that EDCs can affect immune responses upon binding to the hormone receptors expressed on the surface of or in immune cells ^14, 15^ and cause abnormalities relevant to pathology is poorly recognized or not recognized at all. The primary focus of ENDOMIX on the emerging field of immunotoxicity is not limited to immune-related diseases, but applies to a wide range of pathophysiological conditions. Acute or chronic exposure to EDC chemicals and mixtures can impact the immunome, a term used to comprise the genes and proteins that constitute the immune system ^16^, and also affect the frequency, phenotype and functionality of immune cells, with different effects depending on individual predisposition, time and duration of exposure. However, whether and how exposure to chemicals, and in particular EDCs and chemical mixtures of concern, is associated with immune-mediated pathologies has not been studied in depth. The vision of ENDOMIX is to fully elucidate and understand the overall effects of complex EDC mixtures and the underlying mechanisms,

Our strategy is to cover the entire knowledge chain to achieve this ambitious goal ( Figure 1). In a first step candidate EDC mixtures relevant for endocrine disruption, immunotoxicity and immunomodulation will be identified using a previously developed prioritization workflow for neurotoxic chemicals based on simulated worst case exposure, high-throughput toxicokinetics models and high-throughput toxicity data with data-gap filling through toxicity prediction models ^17^. The identified potential mixture effect contributors will be searched for in population-based blood exposure data from a large collection of 16 cohorts and cohort consortia European cohorts including target and non-target analysis using high-resolution mass spectrometry (HRMS) data. Based on this information, complex EDC mixtures will be reconstituted in the laboratory at the concentration ratios as they were detected in human blood. The reconstituted mixtures will be used to investigate their effects at the level of immune cells and target organs. The concentration ratios of the chemicals in the mixtures correspond to the concentrations found in the cohort samples, but the overall concentration is higher to be able to measured but the results will be extrapolated down to the real-life concentrations. Tissue barrier systems, organ-on-a-chip-approaches and zebrafish embryo models behavioral assays will be utilized to understand EDCs' impact on defined endpoints. By combining the gained knowledge on defined associations between exposures and health outcomes and identified cell- and organ-dependent mechanisms, we will continue using cohort samples and model systems to understand causality between these exposures and defined health outcomes and identify underlying mechanisms. Finally, at the end of the project, we will be able to provide evidence-based recommendations to regulators and policy makers, inform the public and contribute to improved risk assessment.

## ENDOMIX objectives

ENDOMIX aims at unravel the overall immunotoxic or immunomodulatory impact of EDC mixtures leading to adverse health outcomes. **The overarching objective of ENDOMIX is to deliver new information and concepts about the immune-mediated health impact of EDC mixtures, and their underlying biological mechanisms, which will build the basis to elaborate on approaches that help minimize EDC exposures in the future.**

Mixtures identified as potentially concerning will be tested in *in vitro* and *in vivo* assays, including primary immune cells, organoids, alternative models such as *Caenorhabditis elegans* and zebrafish embryos, and classic mouse models to uncover mechanistic insights. Barrier models will be used to determine the transport and targets of EDC mixtures. Computational approaches will bolster associations between multiple exposures and health outcomes.

Our objective is to provide clear scientific evidence on exposure scenarios, co-exposures' effects (including lifestyle and socioeconomic factors), target cells and organs, and mechanistic pathways. ENDOMIX will integrate harmonised data on EDC exposures, immune markers, and health outcome endpoints from multiple European cohorts to accelerate this integrative approach. Additionally, we will identify novel bioactive EDCs through predictive exposure and toxicokinetic modelling, combined with data mining from high-throughput screening (HTS) bioassays, with an emphasis on immunotoxicity and immunomodulation, to prioritise drivers of mixture effects.

## Objective 1: Identification of complex EDC mixtures in European populations

Existing knowledge about EDC impact on health mostly came from studies of single chemicals and previous experimental design is often insufficient as they focus on known chemicals only. People are exposed to a wide range of EDCs that can potentially interact and will certainly act together in mixtures. Therefore, it is vital to improve our understanding of the effects of exposure to real-life EDC scenarios at different life stages. Additionally, the problem is that there are potentially thousands of chemicals in our blood and there are hundreds of chemicals that potentially cause endocrine disruption and immunomodulation, but they were never systematically matched to identify chemicals that are mixture effect drivers. ENDOMIX cutting edge and novel approach is that we will simulate mixtures of potential concern by data mining of the toxicology literature, simulating mixture exposure with help of high-throughput toxicokinetic modelling and mixture effect models. This approach has so far led to >7000 candidate chemicals out of a starting list of 100,000 chemicals that could potentially be contributing to mixture effects. ENDOMIX will derive complex EDC mixtures and health outcomes in European populations using an exceptional wealth of data and biosamples analysis by systematic *in silico* prioritization leveraging toxicology literature, high-throughput toxicity & exposure methods and mixture effect models. *In silico* mixtures predictions will be augmented with existing blood biomonitoring data through searching for candidate chemicals of concern data from cohort samples within the consortium. Newly detected bioactive compounds will be confirmed in a small subset of blood samples from the cohorts before complex chemical mixtures are designed for testing in the HTS assays.

ENDOMIX will design *in vitro* studies to analyse endpoints that are relevant for immunotoxicants and associated mediators of health outcomes as defined in the cohort studies. Based on key characteristics (KC) of immunotoxicity, we will select adequate HTS assays addressing relevant key events of endocrine disruption and immunomodulation as a comprehensive apical endpoint of immunotoxicity in innate and adaptive immune cells. These assays are suitable for screening large numbers of chemicals, mixtures and ultimately even extracts from blood samples of the cohorts. The large number of screened chemicals and mixtures in the HTS assays will then be used to develop a smaller number of representative mixtures of immunotoxicants that will be tested in more specialised and more complex bioassays, primary cell cultures and *in vivo* model systems.

**Figure 2.**
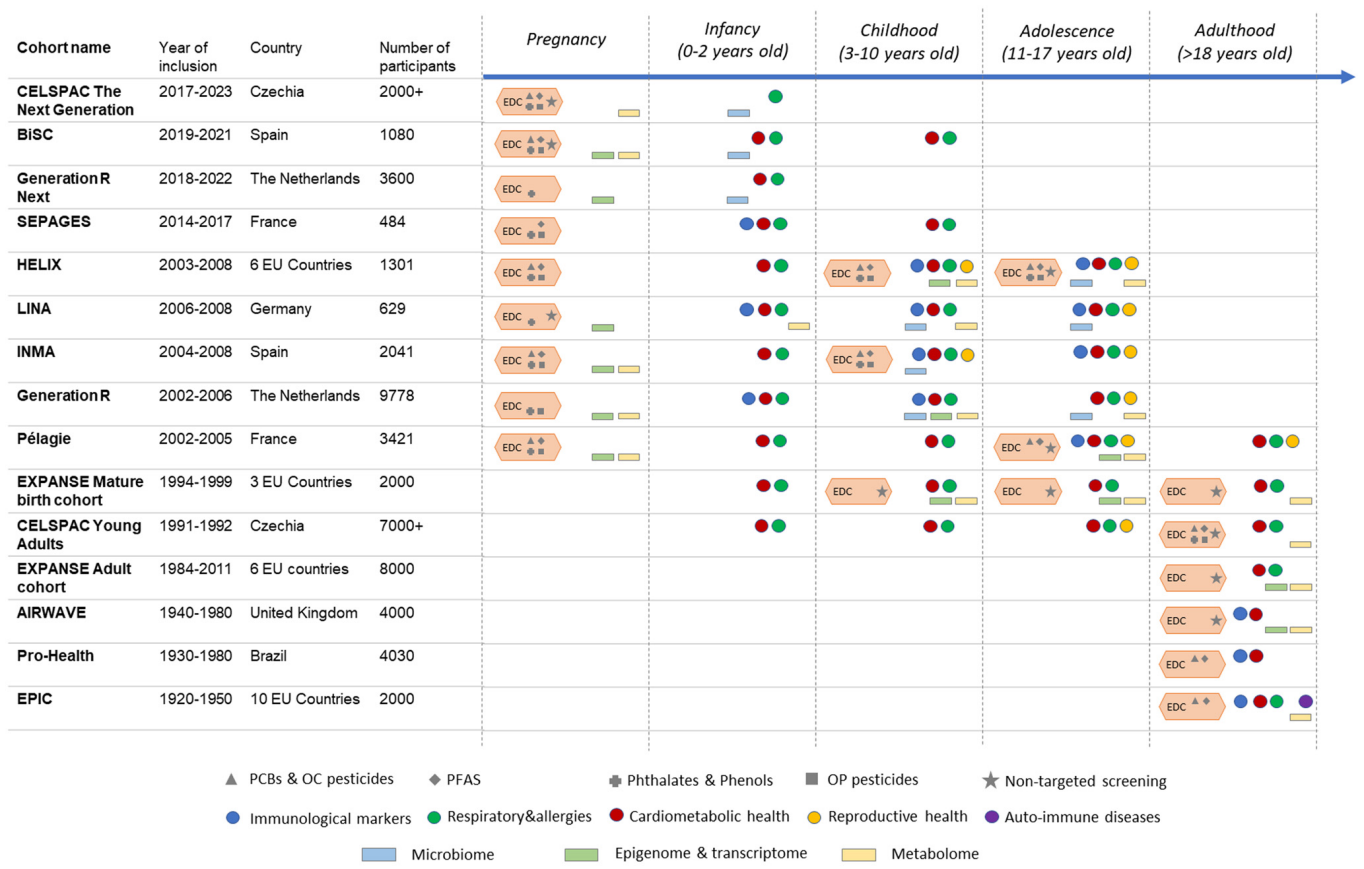
Overview of the observational studies involved in ENDOMIX. Cohort study name, life course timepoints (pregnancy, infancy, childhood, adolescence, and adulthood) and illustration with coloured signs representing the availability of chemical exposure data measured with targeted analysis throughout the life course is shown. Further, available microbiome, epigenome, transcriptomics, and metabolomics data are indicated.

Our strategy starts with population-based studies that include many cohorts covering the life course (prenatal, perinatal, infancy, childhood, adolescence, adulthood) and comprising regions across Europe and beyond with very detailed data (Participating cohorts, Figure 2). ENDOMIX not only brings together large multiple existing cohorts with already measured EDCs in biosamples, but its novelty lies in the identification and study of EDC as mixtures and the focus on the immune-mediated effects of EDCs. In addition to the identification of new mixture contributors, ENDOMIX will use existing targeted screening data of a number of EDCs: polychlorinated biphenyls, organochlorine pesticides, per- and polyfluorinated compounds, metals, parabens, phthalates, phenols, and organophosphate pesticides. Further, for cohorts with limited availability of EDC data, such as for relatively rare outcomes like autoimmune diseases, we will proceed to quantify chemicals suspected to contribute to immunotoxic mixture effects, such as polychlorinated biphenyls, organochlorine pesticides, and per- and polyfluorinated compounds. Another strength is the covering of various life stages allowing the study of different windows of susceptibility to EDCs including early-life and adolescence, two major sensitive periods subject to important endocrine and physiological changes. We expect that EDC mixtures may vary over time due to changes in EDC use, exposure sources, and policies, and across different populations depending on determinants such as age, sex, socioeconomic status, and lifestyle habits. In addition to most of the identification of new mixture contributors, involved cohorts benefit from exposure and health outcome data already harmonised in the context of previous or ongoing EU-funded projects including the LifeCycle project ^18^ and projects that are part of the European Human Exposome Network (EHEN, including ATHLETE ^19^, LongITools ^20^ and Expanse ^21^. ENDOMIX will use existing targeted screening data of a number of EDCs: polychlorinated biphenyls, organochlorine pesticides, per- and polyfluorinated compounds, metals, parabens, phthalates, phenols, and organophosphate pesticides.

## Objective 2: Associations between exposure to EDC mixtures, occurrence of allergies and respiratory, cardiometabolic and reproductive health as well as autoimmunity

Mixtures that occur in real life will be tested for immunotoxic effects from HTS using *in vitro* bioassays and predictive exposure models. After identifying mixtures of concern and testing their effects in simple bioassays, we will examine their potential determinants including socioeconomic, environmental and lifestyle-related factors and their link with several important health outcomes, of which; the choice was based on the potential involvement of the immune system as a mediator of disease development. Immune cells can be targeted by EDCs, are ubiquitous in the human body, can traffic to target organs and if dysfunctional, they are able to initiate, trigger or perpetuate disease. Health outcomes to be studied within ENDOMIX therefore are (1) allergies & respiratory health (eczema, atopic dermatitis, allergic rhinitis, wheezing, asthma, lung function, COPD), (2) cardiometabolic & cardiovascular health (body mass index, waist circumference, adiposity, dyslipidemia, insulin resistance, diabetes, stroke, acute myocardial infarction) (3) reproductive health (age at menarche, age at voice break, Tanner stages, menstrual cycle characteristics, sex hormone levels), and (4) autoimmune diseases (rheumatoid arthritis, ulcerative colitis, Crohn´s disease). The health outcomes will be studied at different age periods, from childhood to adulthood (e.g., from early changes in blood pressure to the onset of cardiovascular diseases) in cohorts and disease models. We will additionally explore how EDCs may be associated with overall health by analysing their potential effects on more than one health outcome, an approach particularly relevant for public health to identify the EDC mixtures that contribute to multimorbidity.

The association of EDCs with the health outcomes may be linked to central immunotoxicity processes with a focus on inflammation. ENDOMIX will identify immune markers focused on inflammation using a proteomics approach and will examine their relation with EDC exposure. Immune markers focused on inflammation will comprise cytokines, chemokines, their respective receptors and a wide range of other immune proteins that will be identified through novel proteomic analyses using Olink® inflammation panels. Many biological pathways. metabolic perturbations may serve as biological pathways involved in immune-related effects of EDCs on human health. ENDOMIX will make use of available epigenetic, microbiomic, and endogenous metabolomic data of participants of the human cohorts. By doing so, we will offer insights into the relationships of exposure to EDCs, immune markers and DNA methylation, microbiome, and metabolome at multiple life stages and their association with human health outcomes.

Studying EDC mixtures in epidemiology raises methodological challenges due to the high correlations between different EDCs and the high dimension of data to be considered (tens to hundreds of chemicals). ENDOMIX will overcome these challenges by using advanced statistical methods suitable for chemical mixtures. These include unsupervised dimensionality reduction and clustering methods that will allow the identification of specific profiles of exposure independently of their health effects, and supervised techniques (e.g., Bayesian Kernel Machine Regression, Quantile G-computation) to estimate both the overall effect of the EDC mixture on disease risk and the contribution of each individual compound to the mixture effect. Another statistical challenge is the integration of immunological markers as potential intermediate endpoints between EDC exposure and health. We will perform mediation analysis in a high dimensional context to disentangle the direct and indirect effect of EDCs through changes in immunological markers. To synthesize information across different biological layers, we will use a multi-omics framework using graphical networks and multi-block approaches, such as two-way orthogonal PLS (O2PLS) and multi-omics factor analysis (MOFA), to integrate the information from EDC exposures, omics layers, possible sex differences and disease outcome.

## Objective 3: Impact of prioritized EDC mixtures on the immunome, innate and adaptive immune cell functionality and target organs

To investigate the direct effects of priority EDC mixtures on the immunome, innate and adaptive immune cells’ frequency, phenotype and functionality, we will first characterise the imunome and which cell subtypes are the most affected by EDC mixtures by mass cytometry, based on CyTOF® (cytometry by time of flight) technology. The unique metal labelling of antibodies allows the simultaneous identification of up to 50 protein markers in one cell with the further advantage of no background signal compared to other methods. After identifying the cell subtypes affected the most, more specific immunoassays involving innate and adaptive immune cell populations will be employed to test the impact of chemicals. Moreover, cytokine-bead arrays will be applied to determine cytokines in monocyte, dendritic cell, T and B cell culture supernatants to gain further insights into cytokine secretion patterns ^22^. For T cells, maturation and differentiation assays will be conducted under chemical treatment. For B cells, alterations in antibody production (Ab) capacity (e.g. IgG, IgM) will be determined under the impact of EDC mixtures. Together, these assays will provide detailed information about EDC effects on immune cell differentiation, maturation, plasticity and functionality and will thus increase our knowledge on chemical drivers of acute and chronic diseases. Finally, gene expression analyses using semi-high-throughput qPCR and omics approaches will be employed in chemical-exposed immune cell populations to gain further insights in altered intracellular pathways.

**Figure 3.**
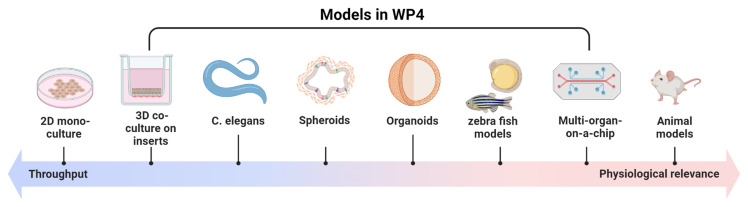
Models used in ENDOMIX. Different models are used in ENDOMIX to study the impact of endocrine disrupting chemical (EDC) mixtures in tissue barriers, cell-cell interaction and whole organisms.

Further, barrier and organ *in vitro* models including organoids will unravel how EDCs behave within tissues, and how EDC mixtures cross barriers and if immune cells work as messengers or mediators of organ toxicity. The models were carefully chosen to be meaningful regarding the health endpoints studied in cohorts. Besides using human *in vitro* models, as described above, we will employ innovative models that faithfully mimic the organs of interest and human response ( Figure 3).

Here, barrier models, co-culture and 3D models, including organoids and organ-on-a-chip models that are already published and running in the laboratories of the ENDOMIX partners, will be used. When adequate or possible, immune cells will be combined with the cell systems. These systems include: *in vitro* lung barrier ^23, 24^, *in vitro* blood-brain-barrier ^25, 26^, *in vitro* placenta barrier ^27^ and *in vitro* intestinal barrier ^28, 29^.

Further, for target organ models, we use lung organoids ^30^, *in vitro* heart spheroids ^31, 32^, intestinal organoids ^33, 34^ and liver spheroids ^35, 36^. Immune cells will be co-cultured to understand EDC effect on their interaction. At the molecular level, we will identify gene and protein expressions of nutrient transporters, regulation of genes related to lipid and glucose metabolism gene and associated transcriptional factors, and perform nuclear receptor transactivation assays. Established gene and protein signatures indicative of triglyceride accumulation ^37^ will be transferred to further models to derive characteristic and predictive gene expression patterns *in vitro,* indicative of a certain endocrine effect.

Organoids will be generated from primary samples, like placenta organoids or will be stem-cell derived Multi-organ *in vitro* models will be used to study interactions between different organs (e.g. intestinal-hepatic interaction or liver-adipocyte interaction) and the effect on chemical biotransformation. Because of its defined short life cycle, *C. elegans* can be exploited to test the effect of chemicals on the whole life cycle of an organism (from egg production to development to mature organism). Our innate immune models with *C. elegans* include bacterial infection models and detection of antimicrobial proteins and peptides. EDC mixtures and individual chemicals will be tested on development and innate immune responses by *C. elegans* ^38, 39^. Moreover, a zebrafish embryo model will be used to complement *in vitro* approaches as *C. elegans* to evaluate the impact of EDC on the outcome of metabolic liver diseases and on related intestine-liver axis disruptions including alterations of microbiota and immune cell functions. The zebrafish embryo model represents a 3R-compliant ^40^, alternative model that, up to 5 days post-fertilization (dpf), is considered to be a non-protected life stage, according to EU regulations ^41^.

There is a gap in the availability of complex, higher-throughput immunotoxicity screening systems. We propose that the zebrafish embryo model can fill this gap, particularly for identifying innate immunotoxicants, as the innate immune system develops in isolation in 5-day-old zebrafish embryos. A new alternative assay to detect innate system immunomodulation will be developed and applied in embryonic zebrafish that quantitates, in parallel, a concentration-response relationship on a diverse suite of ~25 immunomodulatory molecules including markers for phagocytes (neutrophils, monocytes, macrophages), inflammation-related serum proteins (complement, C-reactive protein), antimicrobial peptides (defensins), cell receptors that signal a defensive response (TLRs), cells that release inflammatory mediators (macrophages, mast cells, NK cells), and markers of physical barriers (tight junctions). In addition, automated morphometrics will be applied to quantitate gastrointestinal area which, when increased in the absence of other morphological effects, may indicate intestinal inflammation. Individual chemicals and mixtures developed in this project will be tested in a concentration response format. Transgenic lines labelling neutrophils or macrophages will be used to quantify immune cell migration into the gastrointestinal (GI) tract.

Critical periods of exposure and possible sex differences are also considered in ENDOMIX´s design as for example placenta organoids and immune cells from both sexes are employed to represent exposure that affects not only mother and offspring in a transgenerational approach but also sex-related disease development. The use of alternative models including transgenic zebrafish with labelled macrophages and *C. elegans* as a model of dysbiosis will provide valuable data on EDC impact and immune-mediated mechanisms and thereby greatly reduce the use of vertebrate animal experiments.

## Objective 4: Establishment of causality of EDC exposure on health outcomes

The direct causality between exposure to EDC mixtures and deviations from normal healthy development or disease outcomes needs to be confirmed to deliver evidence for improved chemical regulations. ENDOMIX will utilize the information obtained in Objectives 1–3 to design studies addressing causality of EDC exposures on health outcomes. The models will further consider the EDC association with health outcomes through their impact on immune cells and target organs. These approaches include deep characterisation in cohort samples using omics-based biomarkers and the effect and perinatal exposure using transgenerational *in vivo* models. Experimental mouse models will be used whenever *in vitro* or alternative models cannot answer the scientific question. Mixtures identified previously will be tested in well-defined transgenerational 3R-conform mouse models to verify adverse effects observed in human cohorts and in *in vitro* models. Control treatment will depend on the nature of the defined mixtures. These models may include: asthma in the offspring of exposed mothers, rheumatoid arthritis in offspring of exposed mothers and will include mechanistic studies ^42– 44^. As for *reproductive health,* we will study potential EDC effects on the reproductive performance of female and male offspring of EDC-exposed dams (same exposure protocol as for the other disease models) ^45, 46^. To assess puberty onset in female offspring, we will assess vaginal opening and oestrous cycle analyses, and as ovarian follicle maturation analyses ^47, 48^. For male offspring, sperm analyses will provide insights into their fertility status ^49^.

## Objective 5: Engagement with regulators and providing guidance on exposure to the European community

The European public needs access to well-founded information on the effects of complex mixtures of EDCs to avoid disinformation. Citizens need to be empowered to self-regulate and reduce their exposure to EDCs and mixtures of concern in everyday life to increase the number of healthy life years. ENDOMIX will provide guidance on exposure to EDCs and mixtures and information about potential adverse effects and lay the ground work for regulatory changes and preventive measures for citizens, regulators, and relevant stakeholders within the healthcare value chain. The circle will be closed by using the generated information to develop action plans and recommendations to address priority mixtures of concern that impact health and proposing a more harmonized battery of risk assessment with specific toxicological endpoints and well-defined biomarkers.

To combine information from experimental and epidemiological studies conducted in ENDOMIX and existing and future literature, we will adopt a Weight-of-Evidence (WoE) approach. These approaches are commonly used in authoritative risk assessment programs by organizations such as the WHO, EU, and US-EPA ^33, 50^. In addition, we will utilize novel text mining methodologies to create knowledge graphs that capture the relevant complex biomechanistic insights. Specifically, we will employ the Integrated Network and Dynamical Reasoning Assembler (INDRA) Reach (Reading and Assembling Contextual and Holistic Mechanisms from Text) environment, which uses automated large-scale reading of scientific literature, natural language processing (NLP) approaches, and structured databases to collect and assemble mechanistic understanding of cellular processes and associated health effects ^35^. This approach has already been successfully applied for genotoxic compounds ^51^ and will be extended to EDCs in this study. Through INDRA, mechanistic information from multiple sources will be de-duplicated, standardized, and assembled into sets of statements with associated evidence levels. These Statements can then be used to construct executable rule-based models and a variety of network models, such as knowledge graphs ^51^. We aim to assess the concordance between the traditional WoE approach and generated Statements to provide an expert-led assessment of the validity of this novel computational approach to evidence synthesis”

## Science to policy translation

The scientific insights and knowledge generated by ENDOMIX will be the basis for subsequent application in policy making and development of new regulations. The consortium aims to develop new hands-on research-based guidance and policy options and will provide policy makers and decision makers with new data and evidence-based information. To strengthen the science-policy interface and promote acceptance and uptake of the developed new knowledge base and guidelines, active stakeholder involvement and a thorough consultation process will be ensured through the set-up of a dedicated Stakeholder Board (SHB). The SHB involves members advocating for future policies, scientific advisors, and a citizen representative who provide input early on in the process to ensure their perspectives are considered when developing policy recommendations. ENDOMIX will contribute to the EU’s goal to ensure EU policy making is based on evidence, provide simple, clear and transparent recommendations and involve stakeholders along the knowledge chain in the development. Further, communication and dissemination activities including educational approaches will facilitate science-to-policy translation of the ENDOMIX findings and guidelines.

**Figure 4.**
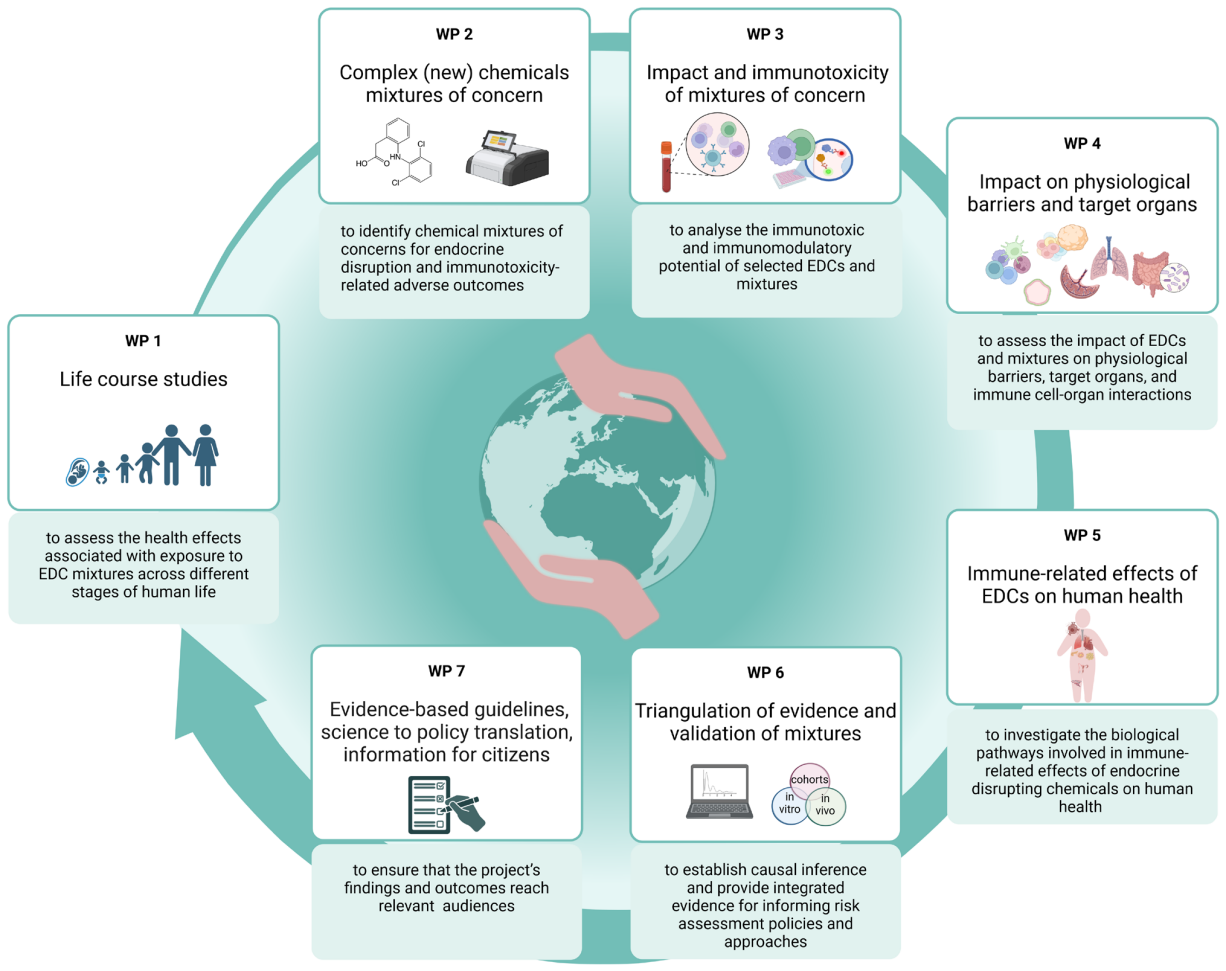
The ENDOMIX concept and workflow. The ENDOMIX project is structured into well-coordinated work packages (WPs) fostering an interdisciplinary approach to understanding the true impact of endocrine disrupting chemical (EDCs) on human health. In addition to the WPs related to the desired research outputs, work dedicated to project coordination and management, clustering activities and ethics requirement is underlying this concept.

The strategy chosen for ENDOMIX to fulfill the objectives is shown in Figure 1 and Figure 4.

Within ENDOMIX, we bring together renowned experts in toxicology, clinical science, environmental immunology, social, environmental and molecular epidemiology, biology, statistics and data science, and risk assessment. Exposure to chemicals can lead to different adverse effects in humans depending on sex, gender, and age. Population studies have shown different exposure patterns in men and women that show a risk factor for diseases such as those related to the immune, reproductive, and metabolic area. Population-based data from the cohorts will be separately analysed for females and males to understand the possible different impacts of different EDC mixtures depending on the sex and on life stages. As for experimental models, ENDOMIX will incorporate the sex difference dimension into the toolbox by using *in vivo* data from animals of both sexes where available for benchmarking the *in vitro* assays and by adapting methods and protocols using cell lines from males as well as from females (where available) as research o has shown the relevance of including both sexes in experiments ^52^. Thus, sex differences in sensitivity, sex-specific variables of biomarkers and sex-specific cut-off values will be considered. From a scientific point of view, sex parameters are specifically considered in our work and attempts to include cell lines and 3D organoids originating from tissues originating from males and females will be made wherever possible. ENDOMIX has already designed organoid studies to contemplate sex differences between female and male, this is particularly relevant for placenta organoids, where the origin of the tissue is often overlooked. Models relying on available cell lines may be a limiting factor for some of the outcomes. Molecular markers are available to determine sex and can be used for retrospective analyses of sex differences. In the epidemiological studies, the models will be adjusted for sex or stratified by sex where applicable.

In conclusion, the ENDOMIX project aims to unravel the complexity of exposure to EDCs and their impact on human health. In particular, ENDOMIX aims at contributing to the understanding of immune-mediated health outcomes and providing comprehensive insights into the biological mechanisms linking EDC exposure to adverse health outcomes, by incorporating multi-omics data, including immunomics, epigenomics, transcriptomics, metagenomics, and metabolomics so to enhance our understanding of the molecular pathways affected by EDCs. By employing already existing cohorts covering different life phases, ENDOMIX can explore the effects of early life EDC exposure and its influence on long-term health trajectories. The knowledge generated by ENDOMIX will inform evidence-based policy-making aimed at reducing EDC exposure, mitigating associated health risks, raising public awareness about the risks of EDC exposure and promoting health-promoting behaviours.

## Disclaimer

The views expressed in this article are those of the author(s). Publication in Open Research Europe does not imply endorsement of the European Commission.

## Ethics and consent

Ethical approval and consent were not required for writing this project summary. The ENDOMIX project itself will be conducted on existing prospectively collected data from different European cohort studies. All participating cohorts have been approved by the relevant ethics boards and informed consent was obtained for all participants.

All research that involves human data is conducted under the rules and legislation in place, including the Declaration of Helsinki, the IHC guideline for Good Clinical Practice, as well as the ISO guidelines on good clinical practice ISO 14155:2020, the European Directive 2001/20/EC on Good Clinical Practice, and the EU General Data Protection Regulation (2016/679).

## Data Availability

No data are associated with this article

## References

[1] Armocida B Monasta L Sawyer S : Burden of non-communicable diseases among adolescents aged 10–24 years in the EU, 1990–2019: a systematic analysis of the Global Burden of Diseases Study 2019. *Lancet Child Adolesc Health.* 2022;6(6):367–383. 10.1016/S2352-4642(22)00073-6 35339209 PMC9090900

[2] Hameed A Farooq T Shabbir S : Role of pharmaceuticals as EDCs in metabolic disorders. *Endocrine Disrupting Chemicals-induced Metabolic Disorders and Treatment Strategies*.2021;357–365. 10.1007/978-3-030-45923-9_21

[3] Bergman A Heindel JJ Kasten T : The impact of endocrine disruption: a consensus statement on the state of the science. *Environ Health Perspect.* 2013;121(4):A104–6. 10.1289/ehp.1205448 23548368 PMC3620733

[4] Martin O Scholze M Ermler S : Ten years of research on synergisms and antagonisms in chemical mixtures: a systematic review and quantitative reappraisal of mixture studies. *Environ Int.* 2021;146: 106206. 10.1016/j.envint.2020.106206 33120228

[5] Bansal A Henao-Mejia J Simmons RA : Immune system: an emerging player in mediating effects of endocrine disruptors on metabolic health. *Endocrinology.* 2018;159(1):32–45. 10.1210/en.2017-00882 29145569 PMC5761609

[6] World Health Organization: Global assessment of the state-of-the-science of endocrine disruptors.International Program on Chemical Safety,2002. Reference Source

[7] Bergman A Heindel JJ Jobling S : State of the science of endocrine disrupting chemicals 2012. World Health Organization;2013. Reference Source

[8] Gore AC Chappell VA Fenton SE : EDC-2: the Endocrine Society's second scientific statement on Endocrine-Disrupting Chemicals. *Endocr Rev.* 2015;36(6):E1–E150. 10.1210/er.2015-1010 26544531 PMC4702494

[9] Meyer A Sandler DP Beane Freeman LE : Pesticide exposure and risk of rheumatoid arthritis among licensed male pesticide applicators in the agricultural health study. *Environ Health Perspect.* 2017;125(7): 077010. 10.1289/EHP1013 28718769 PMC5744649

[10] Kahn LG Philippat C Nakayama SF : Endocrine-Disrupting Chemicals: implications for human health. *Lancet Diabetes Endocrinol.* 2020;8(8):703–718. 10.1016/S2213-8587(20)30129-7 32707118 PMC7437820

[11] United Nations Environment Programme: UNEP 2013 Annual Report.2014. Reference Source

[12] Fuller R Landrigan PJ Balakrishnan K : Pollution and health: a progress update. *Lancet Planet Health.* 2022;6(6):e535–e547. 10.1016/S2542-5196(22)00090-0 PMC11995256 35594895

[13] Popescu M Feldman TB Chitnis T : Interplay between endocrine disruptors and immunity: implications for diseases of autoreactive etiology. *Front Pharmacol.* 2021;12: 626107. 10.3389/fphar.2021.626107 33833678 PMC8021784

[14] Cunningham M Gilkeson G : Estrogen receptors in immunity and autoimmunity. *Clin Rev Allergy Immunol.* 2011;40(1):66–73. 10.1007/s12016-010-8203-5 20352526

[15] Buskiewicz IA Huber SA Fairweather D : Chapter 4 - Sex hormone receptor expression in the immune system. In: Gretchen NN, Megan MM, editors. *Sex Differences in Physiology*. Boston: Academic Press;2016;45–60. 10.1016/B978-0-12-802388-4.00004-5

[16] Biancotto A McCoy JP : Studying the human immunome: the complexity of Comprehensive Leukocyte Immunophenotyping. *High-Dimensional Single Cell Analysis: Mass Cytometry, Multi-parametric Flow Cytometry and Bioinformatic Techniques.* 2013;23–60. 10.1007/82_2013_336 PMC4184245 23975032

[17] Braun G Escher BI : Prioritization of mixtures of neurotoxic chemicals for biomonitoring using high-throughput toxicokinetics and mixture toxicity modeling. *Environ Int.* 2023;171: 107680. 10.1016/j.envint.2022.107680 36502700

[18] Jaddoe VWV Felix JF Andersen AMN : The LifeCycle Project-EU Child Cohort Network: a federated analysis infrastructure and harmonized data of more than 250,000 children and parents. *Eur J Epidemiol.* 2020;35(7):709–724. 10.1007/s10654-020-00662-z 32705500 PMC7387322

[19] Vrijheid M Basagaña X Gonzalez JR : Advancing Tools for Human Early Lifecourse Exposome Research and Translation (ATHLETE): project overview. *Environ Epidemiol.* 2021;5(5):e166. 10.1097/EE9.0000000000000166 34934888 PMC8683140

[20] Ronkainen J Nedelec R Atehortua A : LongITools: dynamic longitudinal exposome trajectories in cardiovascular and metabolic noncommunicable diseases. *Environ Epidemiol.* 2022;6(1):e184. 10.1097/EE9.0000000000000184 35169663 PMC8835657

[21] Vlaanderen J De Hoogh K Hoek G : Developing the building blocks to elucidate the impact of the urban exposome on cardiometabolic-pulmonary disease: the EU EXPANSE project. *Environ Epidemiol.* 2021;5(4):e162. 10.1097/EE9.0000000000000162 34414346 PMC8367039

[22] Fischer F Ermer MR Howanski J : Single and mixture effects of Bisphenol A and Benzophenone-3 on *in vitro* T helper cell differentiation. *Chem Biol Interact.* 2024;395: 111011. 10.1016/j.cbi.2024.111011 38653352

[23] Kovar L Wien L Selzer D : In Vitro-In Silico modeling of Caffeine and Diclofenac Permeation in static and fluidic systems with a 16HBE lung cell barrier. *Pharmaceuticals (Basel).* 2022;15(2):250. 10.3390/ph15020250 35215362 PMC8876625

[24] Kohl Y Müller M Fink M : Development and characterization of a 96–well exposure system for safety assessment of nanomaterials. *Small.* 2023;19(23): e2207207. 10.1002/smll.202207207 36922728

[25] Danz K Höcherl T Wien SL : Experimental comparison of primary and hiPS-based *In Vitro* Blood-Brain Barrier models for pharmacological research. *Pharmaceutics.* 2022;14(4):737. 10.3390/pharmaceutics14040737 35456571 PMC9031459

[26] Rohde F Danz K Jung N : Electrospun scaffolds as cell culture substrates for the cultivation of an in vitro blood-brain barrier model using human induced pluripotent stem cells. *Pharmaceutics.* 2022;14(6): 1308. 10.3390/pharmaceutics14061308 35745880 PMC9231001

[27] Stojanovska V Arnold S Bauer M : Characterization of three-dimensional trophoblast spheroids: an alternative model to study the physiological properties of the placental unit. *Cells.* 2022;11(18): 2884. 10.3390/cells11182884 36139458 PMC9497053

[28] Stock V Bohmert L Coban G : Microplastics and nanoplastics: size, surface and dispersant - What causes the effect? *Toxicol In Vitro.* 2022;80: 105314. 10.1016/j.tiv.2022.105314 35033651

[29] Paul MB Schlief M Daher H : A human Caco-2-based co-culture model of the inflamed intestinal mucosa for particle toxicity studies. *In vitro Models.* 2023;2(1):43–64. 10.1007/s44164-023-00047-y 39872873 PMC11756451

[30] Mason RJ Dobbs LG : Alveolar epithelium and pulmonary surfactant. *Murray and Nadel's Textbook of Respiratory Medicine.* 2016;1:134–149. e5. 10.1016/B978-1-4557-3383-5.00008-7

[31] Fischer B Meier A Dehne A : A complete workflow for the differentiation and the dissociation of hiPSC-derived cardiospheres. *Stem Cell Res.* 2018;32:65–72. 10.1016/j.scr.2018.08.015 30218895

[32] Lauschke K Rosenmai AK Meiser I : A novel human pluripotent stem cell-based assay to predict developmental toxicity. *Arch Toxicol.* 2020;94(11):3831–46. 10.1007/s00204-020-02856-6 32700165 PMC7603451

[33] Vandenberg LN Agerstrand M Beronius A : A proposed framework for the systematic review and integrated assessment (SYRINA) of endocrine disrupting chemicals. *Environ Health.* 2016;15(1): 74. 10.1186/s12940-016-0156-6 27412149 PMC4944316

[34] Klooster JPT Bol-Schoenmakers M van Summeren K : Enterocytes, fibroblasts and myeloid cells synergize in anti-bacterial and anti-viral pathways with IL22 as the central cytokine. *Commun Biol.* 2021;4(1): 631. 10.1038/s42003-021-02176-0 34045640 PMC8160143

[35] Bachman JA Gyori BM Sorger PK : Automated assembly of molecular mechanisms at scale from text mining and curated databases. *Mol Syst Biol.* 2023;19(5): e11325. 10.15252/msb.202211325 36938926 PMC10167483

[36] Rose S Ezan F Cuvellier M : Generation of proliferating human adult hepatocytes using optimized 3D culture conditions. *Sci Rep.* 2021;11(1): 515. 10.1038/s41598-020-80019-4 33436872 PMC7804446

[37] Lichtenstein D Mentz A Sprenger H : A targeted transcriptomics approach for the determination of mixture effects of pesticides. *Toxicology.* 2021;460: 152892. 10.1016/j.tox.2021.152892 34371104

[38] Bhalla D Steijaert MN Poppelaars ES : DARTpaths, an *in silico* platform to investigate molecular mechanisms of compounds. *Bioinformatics.* 2023;39(1): btac767. 10.1093/bioinformatics/btac767 36477801 PMC9825785

[39] van der Voet M Teunis M Louter-van de Haar J : Towards a reporting guideline for developmental and reproductive toxicology testing in *C. elegans* and other nematodes. *Toxicol Res (Camb).* 2021;10(6):1202–10. 10.1093/toxres/tfab109 34950447 PMC8692742

[40] Hubrecht RC Carter E : The 3Rs and humane experimental technique: implementing change. *Animals (Basel).* 2019;9(10):754. 10.3390/ani9100754 31575048 PMC6826930

[41] Strähle U Scholz S Geisler R : Zebrafish embryos as an alternative to animal experiments--a commentary on the definition of the onset of protected life stages in animal welfare regulations. *Reprod Toxicol.* 2012;33(2):128–32. 10.1016/j.reprotox.2011.06.121 21726626

[42] Buchenauer L Haange SB Bauer M : Maternal exposure of mice to glyphosate induces depression- and anxiety-like behavior in the offspring via alterations of the gut-brain axis. *Sci Total Environ.* 2023;905: 167034. 10.1016/j.scitotenv.2023.167034 37709081

[43] Buchenauer L Junge KM Haange SB : Glyphosate differentially affects the allergic immune response across generations in mice. *Sci Total Environ.* 2022;850: 157973. 10.1016/j.scitotenv.2022.157973 35963408

[44] Fischer F Kretschmer T Seifert P : Single and combined exposures to Bisphenol A and Benzophenone-3 during early mouse pregnancy have differential effects on fetal and placental development. *Sci Total Environ.* 2024;922: 171386. 10.1016/j.scitotenv.2024.171386 38431166

[45] Santamaria CG Meyer N Schumacher A : Dermal exposure to the UV filter Benzophenone-3 during early pregnancy affects fetal growth and sex ratio of the progeny in mice. *Arch Toxicol.* 2020;94(8):2847–2859. 10.1007/s00204-020-02776-5 32430675

[46] Müller JE Meyer N Santamaria CG : Bisphenol A exposure during early pregnancy impairs uterine Spiral Artery remodeling and provokes intrauterine growth restriction in mice. *Sci Rep.* 2018;8(1): 9196. 10.1038/s41598-018-27575-y 29907759 PMC6003928

[47] Gaytan F Morales C Leon S : Development and validation of a method for precise dating of female puberty in laboratory rodents: the Puberty Ovarian Maturation Score (Pub-Score). *Sci Rep.* 2017;7: 46381. 10.1038/srep46381 28401948 PMC5388887

[48] Chen Y Liu Q Liu R : A prepubertal mice model to study the growth pattern of early ovarian follicles. *Int J Mol Sci.* 2021;22(10):5130. 10.3390/ijms22105130 34066233 PMC8151218

[49] Jeschke L Santamaria CG Meyer N : Early-Pregnancy Dydrogesterone supplementation mimicking luteal-phase support in ART patients did not provoke major reproductive disorders in pregnant mice and their progeny. *Int J Mol Sci.* 2021;22(10):5403. 10.3390/ijms22105403 34065597 PMC8161261

[50] Pipal M Wiklund L Caccia S : Assessment of Endocrine Disruptive properties of PFOS: EFSA/ECHA guidance case study utilising AOP networks and alternative methods. *EFSA J.* 2022;20(Suppl 1): e200418. 10.2903/j.efsa.2022.e200418 35634558 PMC9131586

[51] Scholten B Simón LG Krishnan S : Automated network assembly of mechanistic literature for informed evidence identification to support cancer risk assessment. *Environ Health Perspect.* 2022;130(3):37002. 10.1289/EHP9112 35238605 PMC8893280

[52] Miller I Diepenbroek C Rijntjes E : Gender specific differences in the liver proteome of rats exposed to short term and low-concentration Hexabromocyclododecane (HBCD). *Toxicol Res (Camb).* 2016;5(5):1273–1283. 10.1039/c6tx00166a 30090431 PMC6062380

