# Supplementary material for: The ENDOMIX project: an interdisciplinary approach to understanding how real-life chemical mixtures target the immune system to trigger disease
Source: Open Res Eur. 2025 Nov 21;4:271. Originally published 2024 Dec 19. [Version 2] doi: 10.12688/openreseurope.19088.2 (PMC12673258; doi:10.12688/openreseurope.19088.2)
Supplement: Peer review history

# Reviewer report: Reviewer response for version 2

**DOI:** 10.21956/openreseurope.23738.r64836

**Published:** 2025-12-30

**Authors:** David Lopez-Rodriguez

**Affiliations:** 1 Predictive Modelling, Pharma Research and Early Development (pRED), F. Hoffmann-La Roche Ltd, Basel, Switzerland

EDCs are clearly a growing concern for the environment, wildlife, and humans around the globe. Despite this, most of the literature in the field has focused on evaluating the same molecules and endpoints. As a consequence, there is an urgent need to better understand the wide range of molecules to which we are all exposed, the health consequences of complex EDC mixtures and the assessment of less commonly investigated endpoints such as the immune system. The ENDOMIX project arrives at a very timely moment to help fill critical gaps in knowledge in the field. 

 Please find here a series of comments that may help to further improve the manuscript and the project:

 1) Despite being a large consortium, the project may be overly ambitious, spanning a multiplicity of methods, topics and expected outcomes. For instance, the core of the project relies on the use (and development) of non-standard assays, mixture assessment, the implementation of NAMs, extremely complex experimental designs - often difficult to interpret - and in silico and statistical/mathematical methods. Contributing to the development of guidance within the timespan of this project, promoting regulatory acceptance of such complex and non-standardised methods or supporting decision-making using complex EDC mixture evidence seems —unfortunately— unrealistic. The authors may consider being more specific in their intended outcomes within the science-to-policy context (e.g., contributing to public awareness via press releases, publishing novel methods and contributing to their integration as an OECD guideline, generating new methods for EDC mixture risk assessment or IVIVE).

 2) Following the previous point, this open letter would benefit from a figure schema of the different WPs, their expected outcomes and the broad methodological approaches to be implemented. Additionally, the schema could include an overview of the broad timeline and show how the different WPs will be integrated into a cohesive approach. This figure would provide a more informative big-picture overview of the project than Figure 4, which does not add more value than the section headers in the main text. The schema could also outline clear actionable steps (for instance, for regulatory relevance).

 3) Many biomonitoring projects have been conducted in the past 10–15 years in Europe. One of them, HBM4EU, stands out and I am surprised it is not included in Figure 2. Is there a particular reason for this? If the list is not intended to be exhaustive, this should be indicated in the figure caption. Additionally, we live in an ever-changing chemical environment and pollutans found in the 50s may have nothing to do with the ones we are exposed today. How these aspects are considered in the cohorts authors have selected to define the final mixture? 

 4) From a practical perspective, many different research teams will be working in parallel to achieve ENDOMIX goals. In fact, several ambitious EU-funded projects in the past have struggled to integrate results across partners, ending up by publishing isolated papers within each team’s domain of expertise. A concise and short overview of the project collaboration framework, leadership structure and about the integrative strategy that will be performed would definitely strengthen confidence in the feasibility of the project.

 5) The concept of uncertainty assessment is not mentioned. While I don't expect a detailed methodology in an open letter, it would be worth mentioning the integration of uncertainty evaluation across all proposed models, specially for the extrapolation to health outcomes and to have a clear quantitative impact on decision making.

 6) Objective 6 aims to establish a causal link between EDC exposure and health outcomes. It is unclear how transgenerational in vivo studies will address this question, given that evidence for transgenerational immune effects in humans is largely lacking. Are the authors referring to multigenerational studies rather than germline-mediated effects (true transgenerational effects), which require assessing at least three generations? Unless the intention is to compare germline versus somatic EDC effects (which seems to be out of the scope here), it is not clear how transgenerational studies would support causal associations. Additionally, reproductive transgenerational effects of EDC mixtures have already been investigated and seem outside the scope of this proposal, which focuses primarily on immune outcomes. If the authors intend to retain transgenerational studies, further clarification of the specific endpoints and the definition of causal relationships would be helpful.

 7) Broadly, several ideas across the letter are repeated multiple times. Specifically, Page 5 of the manuscript reiterate the knowledge gaps relating EDCs to immune disorders. In this case, the first column of page 5 could be revised to include actual evidence from human studies, for instance, summarising recent findings in the field, for example. Reducing redundancy would also create space for a short section discussing major methodological or conceptual challenges associated to the project, which would be greatly appreciated. 

 8) Regarding the definition of the chemical mixtures to be evaluated, how will the authors ensure reproducibility across cohorts considering differences in sample types, measurement platforms, time periods, developmental stages, populations, socioeconomic contexts, types of molecules,...?

 9) The term “mixture effect drivers” could be briefly defined.

 10) Statements such as “The vision of ENDOMIX is to fully elucidate and understand the overall effects of complex EDC mixtures and the underlying mechanisms” should be toned down.

 11) Small typo on page 7: “analysisby” -> “analysis by” (only present in the pdf)

Where applicable, are recommendations and next steps explained clearly for others to follow? (Please consider whether others in the research community would be able to implement guidelines or recommendations and/or constructively engage in the debate)

Partly

Does the article adequately reference differing views and opinions?

Partly

Are all factual statements correct, and are statements and arguments made adequately supported by citations?

Yes

Is the rationale for the Open Letter provided in sufficient detail? (Please consider whether existing challenges in the field are outlined clearly and whether the purpose of the letter is explained)

Partly

Is the Open Letter written in accessible language? (Please consider whether all subject-specific terms, concepts and abbreviations are explained)

Yes

Reviewer Expertise:

Endocrine disruption, Neurotoxicity, NAMs, computational toxicology, DART, neuroendocrinology, transgenerational EDC efffects.

I confirm that I have read this submission and believe that I have an appropriate level of expertise to confirm that it is of an acceptable scientific standard, however I have significant reservations, as outlined above.

# Reviewer report: Reviewer response for version 2

**DOI:** 10.21956/openreseurope.23738.r64634

**Published:** 2025-12-02

**Authors:** Jacques Robert

**Affiliations:** 1 University of Rochester Medical Center, Rochester, USA

No further comments

Where applicable, are recommendations and next steps explained clearly for others to follow? (Please consider whether others in the research community would be able to implement guidelines or recommendations and/or constructively engage in the debate)

Partly

Does the article adequately reference differing views and opinions?

Partly

Are all factual statements correct, and are statements and arguments made adequately supported by citations?

Yes

Is the rationale for the Open Letter provided in sufficient detail? (Please consider whether existing challenges in the field are outlined clearly and whether the purpose of the letter is explained)

Yes

Is the Open Letter written in accessible language? (Please consider whether all subject-specific terms, concepts and abbreviations are explained)

Yes

Reviewer Expertise:

Immunotoxicology

I confirm that I have read this submission and believe that I have an appropriate level of expertise to confirm that it is of an acceptable scientific standard.

# Reviewer report: Reviewer response for version 1

**DOI:** 10.21956/openreseurope.20658.r55593

**Published:** 2025-08-29

**Authors:** Josef Köhrle

**Affiliations:** 1 Charité-Universitätsmedizin Berlin, Berlin, Germany

Acute and chronic exposure to environmental chemicals, especially those targeting the endocrine and reproductive systems and crucial neurodevelopmental processes, has been demonstrated to be associated with predisposition, emergence and deterioration of common diseases, especially if exposure occurs during intrauterine development and sensitive developmental windows of the growing and maturing organism. Mechanistic evidence, clear cause-effect relationships between exposure and adverse outcome, established by using informative animal experimental models, and recently accumulating human epidemiological data provide strong support for adverse, frequently irreversible action of chemicals contained in our nutrition, drinking water, cosmetics and consumer products. Limited information is available so far for their adversity on the metabolic and immune system.

 The authors provide a detailed outline of an EU funded project addressing the potential adverse impact of exposures to realistic mixtures of (environmental) chemicals targeting components of the immune system and related diseases. Currently, information on adverse effect of chemicals (not only EDCs) mainly is based on analyses of mechanisms and endpoints for single compound, while realistic exposure scenarios comprise complex mixtures and multiple routes of exposure. The ENDOMIX team intends a multimodal application and combination of several methodological approaches to identify biomarkers accessible by large cohort studies for various advisory policies.

 As the immune system is involved in many crucial processes relevant for health and disease the planned studies might significantly contribute to clarify which chemical mixtures and which of their main components are associated with or even causally involved with adverse outcome of developmental or chronic impact of mixture exposures. Currently, it is still unclear whether underlying mechanisms result in immuno-, neuro-, repro- or endocrino-toxic effects or their combination. Obviously, statements (chapter ENDOMIX objectives) like *“The vision of ENDOMIX is to fully elucidate and understand the overall effects of complex EDC mixtures and the underlying mechanisms. Our strategy is to cover the entire knowledge chain to achieve this ambitious goal ...”, *graphically summarized in Fig.1, are not only ‘ambitious’, but maybe considered as overambitious, boastful and probably unrealistic for the given time frame, team composition and budget restrictions and in the context of many research teams who worldwide work on this complex thematic with serious efforts!

 It is also questionable whether the goal will be achieved that *“ENDOMIX will deliver **evidence-based guidelines **and advice to citizens on improving human health”. *Guidelines is a very clearly defined term in biomedicine. At its best, if successful, ENDOMIX might delineate some **guidance** information for individuals, risk groups, regulatory authorities or policy makers. But consolidation of such information will still need to be confirmed and validated by implementation of properly powered prospective (mid-/long-term) studies which might generate such clinically-relevant evidence for clinical practice and best patient care, including prevention of disease. Authors should be more cautious in their terminology and tone down expectations of the outcome of this challenging project.

 The chosen conventional and innovative state of the art models (8!) for testing the selected mixtures are suitable for the intended purpose and probably will allow identification of novel EDCs and prioritization of drivers of adverse mixture effects with the focus on the immune system. Less clear remains the selection of the chemical mixtures to be tested in the various models based on the exposome data that will be collected from the various cohorts studied. Probably, the inclusion of some already established EDC reference mixtures (e.g. ovarian fluid, amniotic fluid along pregnancy trimesters) that have been studied in recent EU-funded projects would provide benchmarks and allow comparability with existing data. Whether ENDOMIX team will take advantage the extensive HBM4EU exposure information gathered during the last decade and exploit this data remains unclear.  

 ENDOMIX will have access to chemical exposure data from observational studies from several cohorts across Europe which cover various life phases from pregnancy to adulthood (Fig. 2) and will provide rather complex data (microbiome, epigenome, transcriptome and metabolome) on various chemicals contained in real-life mixtures in context of a broad spectrum of disease parameters. Nevertheless, the complexity of this data and the complexity of the immunotoxicity focus of this project will remain a tremendous challenge for its exploitation.

 Whether the 5 main immune system-related health outcome goals of this project (Objective 2) can be achieved with the chosen strategy, selected models and method spectrum provided by the multidisciplinary but very heterogeneously composed team of experts remains to be seen.

 The team also expects to fill the gap between in vitro model systems to the immune system operating in an intact organism (e.g. from stem cell-derived organoids to the intact organism across its life phases) by exploiting the C. *elegans* innate immune response and zebrafish embryo models. As mentioned before this seems more than ambitious in its extent of goals, parameters and endpoints to be analyzed. Couldn't they decide to leave something out and to prioritize and focus on some key aspects instead of presenting a panoptic scenario?

 In objective 4 the ENDOMIX consortium intends to establish causality of EDC mixture exposure on health outcomes and realistically also plans to use experimental mouse models when the chosen in vitro approaches will reach their predictive and informative limits required for extrapolation and interpretation of in vivo conditions. However, they do not elaborate in detail for which diseases which proper mouse models are existing and for which they are still lacking and where their limitations are in context of immunotoxic EDC effects and mechanisms involved.

 The last and 5 ^th^ objective focusses on future ***guidance*** for consumers and the society to avoid and reduce EDC exposure. Are there any strategies, benchmarks, criteria already available how potential endpoints of preventive measures can be established, validated and tested for their applicability and success?

 This project will provide novel insight into the role of immunotoxic effects of EDC exposure during various life phases. It will contribute to a better understanding of the impact, role and interactions of the immune system for developmental processes relevant for the endocrine, reproduction and neurological systems. It can be expected that data accumulated by the EU-funded ENDOMIX consortium can contribute to better **guidance** for reduction of individual, environmental and societal EDC exposure which can only complement EU regulatory measures to stop the production, further distribution and application of identified and suspected endocrine disrupting chemicals (EDCs), which are substance of very high concern (SVHC) like mutagens, carcinogens and reprotoxicants. Probably, several of these chemicals and their mixtures not only act as EDCs but are also neurotoxic and/or immunotoxic, adversely impacting the health of living organisms including humans, especially during development in an irreversible manner.

Where applicable, are recommendations and next steps explained clearly for others to follow? (Please consider whether others in the research community would be able to implement guidelines or recommendations and/or constructively engage in the debate)

Partly

Does the article adequately reference differing views and opinions?

Partly

Are all factual statements correct, and are statements and arguments made adequately supported by citations?

Yes

Is the rationale for the Open Letter provided in sufficient detail? (Please consider whether existing challenges in the field are outlined clearly and whether the purpose of the letter is explained)

Yes

Is the Open Letter written in accessible language? (Please consider whether all subject-specific terms, concepts and abbreviations are explained)

Yes

Reviewer Expertise:

molecular aspects of the thyroid hormone system (biosynthesis, distribution, metabolism, action, regulation); effects of endocrine active compounds (endocrine disruptors) upon the thyroid hormone system; interaction between essential trace elements (iodine, selenium, iron) and the endocrine system; development of analytical methods for endocrine-relevant ligands.

I confirm that I have read this submission and believe that I have an appropriate level of expertise to confirm that it is of an acceptable scientific standard, however I have significant reservations, as outlined above.

# Reviewer report: Reviewer response for version 1

**DOI:** 10.21956/openreseurope.20658.r55598

**Published:** 2025-07-22

**Authors:** Jacques Robert

**Affiliations:** 1 University of Rochester Medical Center, Rochester, USA

This an interesting manuscript that presents the ENDOMIX project, which is an ambitious integrative approach to model and measure how real-life endocrine disrupting chemicals (EDCs) act in mixtures to target the immune system and initiate, trigger or maintain disease. ENDOMIX is also anticipated to allow the identification of biomarkers and patterns of chemical exposures that would be convenient to measure, available for large cohorts and indicative for adverse health outcomes. The manuscript is mainly a description of the ENDOMIX approach and anticipate outcomes. As such, the plan is sufficiently detailed and convincing, although it would be great to see some real data to determine how successful this approach will be. The manuscript would benefit from a short preliminary set of real data using ENDOMIX. Overall, the manuscript is well written. There are a few points listed below that the authors may consider that would improve the manuscript.   

 1) It is unclear how robust the prediction of the potential additive or synergistic effects of different EDCs can be based on their structure. Some clarification would be helpful.  In addition, the possibility of interaction between degradation products of the different EDCs as well as the possible interaction of EDCs and their degradation products with metabolites in human tissues should be considered. Some discussion of this complex issue and potential limitation of ENDOMIX would be welcomed.

 2) An important immune function targeted by EDCs that is unfortunately completely omitted in this manuscript is the defects in immune responses to pathogens leading to increase susceptibility to infectious diseases such as influenza and other bacterial pathogens. There are multiple published data from mouse and the amphibian Xenopus models as well as epidemiologic data. Including EDC impacts on this key function of the immune system is crucial and would markedly strengthen the manuscript.

 3) While zebrafish is mentioned as an alternative model, the Xenopus model is left out, which is unfortunate owing its potential for assessing the direct impacts of EDCs on the development of adaptive immunity as well as the indirect EDC effect on immune system development through the thyroid axis. In addition, the Amphibian Metamorphosis Assay (AMA) is a well-established test to determine if a given toxicant has potential to be a thyroid-disrupting chemical (TDC), and guidelines for the AMA have been endorsed by the US Environmental Protection Agency and Organization for Economic Co-operation and Development (OECD). This makes Xenopus an important element in the regulation of industrial chemicals by government entities.

  4) There is little discussion regarding the validation of the data obtained through ENDOMIX. For such a large approach a systematic validation system should be delineated.

Where applicable, are recommendations and next steps explained clearly for others to follow? (Please consider whether others in the research community would be able to implement guidelines or recommendations and/or constructively engage in the debate)

Partly

Does the article adequately reference differing views and opinions?

Partly

Are all factual statements correct, and are statements and arguments made adequately supported by citations?

Yes

Is the rationale for the Open Letter provided in sufficient detail? (Please consider whether existing challenges in the field are outlined clearly and whether the purpose of the letter is explained)

Yes

Is the Open Letter written in accessible language? (Please consider whether all subject-specific terms, concepts and abbreviations are explained)

Yes

Reviewer Expertise:

Comparative immunology, immunotoxicology.

I confirm that I have read this submission and believe that I have an appropriate level of expertise to confirm that it is of an acceptable scientific standard, however I have significant reservations, as outlined above.

# Response

**Published:** 2025-11-14

**Authors:** Thi Anh Mai Pham

Thank you for your valuable and constructive feedback on our manuscript. We have carefully considered the points raised and prepared a detailed, point-by-point response to each comment, indicating the corresponding changes made in the manuscript and where they can be found in the revised version: 

**This an interesting manuscript that presents the ENDOMIX project, which is an ambitious integrative approach to model and measure how real-life endocrine disrupting chemicals (EDCs) act in mixtures to target the immune system and initiate, trigger or maintain disease. ENDOMIX is also anticipated to allow the identification of biomarkers and patterns of chemical exposures that would be convenient to measure, available for large cohorts and indicative for adverse health outcomes. The manuscript is mainly a description of the ENDOMIX approach and anticipate outcomes. As such, the plan is sufficiently detailed and convincing, although it would be great to see some real data to determine how successful this approach will be. The manuscript would benefit from a short preliminary set of real data using ENDOMIX. Overall, the manuscript is well written. There are a few points listed below that the authors may consider that would improve the manuscript.**

***Author response:*** Thanks very much for this positive feedback. As for real data sets, we prefer at this stage to refrain of including data as this is not a classic manuscript but an open letter and this kind of format does not allow for primary data. Besides, given the complexity of a consortium project, it would be difficult to pick one data set to represent to whole consortium.

**1) It is unclear how robust the prediction of the potential additive or synergistic effects of different EDCs can be based on their structure. Some clarification would be helpful.  In addition, the possibility of interaction between degradation products of the different EDCs as well as the possible interaction of EDCs and their degradation products with metabolites in human tissues should be considered. Some discussion of this complex issue and potential limitation of ENDOMIX would be welcomed.**

***Author response:*** From a public health and regulatory perspective, the central question is whether an endocrine-disrupting chemical (EDC) can exert detrimental effects on human health—regardless of whether the toxicity originates from the parent compound or its degradation products/metabolites.

 In epidemiological studies, the decision to measure either the parent compound or its metabolites is guided by the goal of accurately characterizing participants’ exposure levels. For EDCs that are rapidly metabolized upon entering the human body (e.g., phthalates, organophosphate pesticides), exposure is typically assessed via the quantification of their metabolites. In contrast, for more persistent EDCs (e.g., organochlorines, PFAS), exposure is generally evaluated by measuring the parent compounds directly.

 In whole-organism studies (both epidemiological and in vivo), the organism is exposed to the parent compound, which is subsequently metabolized by endogenous pathways. Consequently, the subject is effectively exposed to both the parent compound and its metabolites, and any observed toxicity may result from either or both forms. It is important to acknowledge, however, that humans may also be directly exposed to certain metabolites, and that interindividual variability in metabolic capacity can lead to exposure misclassification in population studies.

 Greater concern arises in in vitro (and potentially in silico) systems, where the metabolic capacity of the test model may be limited or absent. In such cases, exposing cells solely to the parent compound could yield misleading results—particularly if the toxic effect is mediated by metabolites rather than the parent chemical. To address this limitation, it is advisable to test both the parent compound and relevant metabolites when feasible, ensuring a more comprehensive assessment of potential toxic effects. However, our focus is on mixtures. In complex mixtures, the concentration-additive nature of effects becomes largely independent of chemical structure and synergistic effects less important. This additive hypothesis can be tested by comparing responses from single compounds with those from their mixtures—a strategy that has been shown to hold true within one order of magnitude in previous studies. In the ENDOMIX project, our goal is to analyze real-life mixtures. Therefore, as long as robust exposure data are available for both parent compounds and their transformation products, we will design mixtures that include both and systematically assess their effects.

**2) An important immune function targeted by EDCs that is unfortunately completely omitted in this manuscript is the defects in immune responses to pathogens leading to increase susceptibility to infectious diseases such as influenza and other bacterial pathogens. There are multiple published data from mouse and the amphibian Xenopus models as well as epidemiologic data. Including EDC impacts on this key function of the immune system is crucial and would markedly strengthen the manuscript.**

***Author response:*** We agree with the reviewer that this is highly relevant topic and very much related to the immune system. While infectious were not the focus of the project, basically because the health outcomes of the included cohorts do not specifically deal with infectious diseases, we still have included some aspects in our design. For example, in some in vitro approaches, effects of EDC mixtures will be tested under parallel stimulation with bacterial and viral motifs, namely pathogen-associated molecular patterns (PAMPs) and intracellular molecules associated with cellular damage, called damage-associated molecular patterns (DAMPs). Together, these assays will provide detailed information about EDC effects on immune cell differentiation, maturation, plasticity and functionality and will thus increase our knowledge on chemical drivers of acute and chronic diseases. Moreover, our findings will provide information on how EDCs may alter immune responses against pathogens. For clarification, we have revised the first paragraph under “Objective 3: Impact of prioritized EDC mixtures on the immunome, innate and adaptive immune cell functionality and target organs” providing the explanation mentioned and included three additional references (PMIDs: 33049673, 17355946, 7174118).We agree with the reviewer that the Xenopus model represents a strong system to investigate adaptive immunity and thyroid axis disruption. While the embryonic/larval zebrafish model is able to capture thyroid disrupting chemicals (PMID: 26765085), adaptive immunity can only be assessed in older fish, necessitating lower throughput testing that requires an approved animal use protocol. Here, we opted to complement mammalian testing with rapid, early life stage zebrafish-based screening to identify human-relevant chemicals and mixtures that trigger intestinal inflammation. Future work should consider using a Xenopus or juvenile-based zebrafish systems to study the effects of ENDOMIX mixtures on adaptive immunity development and function.

**3) While zebrafish is mentioned as an alternative model, the Xenopus model is left out, which is unfortunate owing its potential for assessing the direct impacts of EDCs on the development of adaptive immunity as well as the indirect EDC effect on immune system development through the thyroid axis. In addition, the Amphibian Metamorphosis Assay (AMA) is a well-established test to determine if a given toxicant has potential to be a thyroid-disrupting chemical (TDC), and guidelines for the AMA have been endorsed by the US Environmental Protection Agency and Organization for Economic Co-operation and Development (OECD). This makes Xenopus an important element in the regulation of industrial chemicals by government entities.**

***Author response:* **We appreciate the reviewer’s suggestion and acknowledge that Xenopus model offers advantages for thyroid hormone disruption-related investigations, nevertheless it is not a common model for studying human immunotoxicity, which is the primary focus of ENDOMIX project. In contrast, zebrafish have been widely adopted as a relevant and suitable model for immunotoxicity research, namely for targets within the innate immunity, as shown in recent literature (e.g. reviews by Zhao et al., 2024, doi: 10.1016/j.ecoenv.2024.116023; Franza et al., 2024, doi: 10.3390/ijms252212008). Moreover, the Amphibian Metamorphosis Assay (AMA) based on Xenopus laevis falls outside the general scope of our project, which focuses namely on the development and implementation of 3R (Replacement, Reduction, Refinement) -compliant approaches complemented by limited in vivo testing using specific mammalian models with direct relevance for human immune health. Unlike the involved in vitro methods and the Fish Embryo Toxicity (FET) assay, the AMA does not align with the 3Rs strategy due to its longer exposure duration of 21 days. Furthermore, AMA primarily focuses on metamorphosis as an endpoint, which is not directly relevant to human health outcomes. Therefore, we chose to include immune disruption-related endpoints in zebrafish embryos as a suitable model within the context of our study. 

**4) There is little discussion regarding the validation of the data obtained through ENDOMIX. For such a large approach a systematic validation system should be delineated.**

***Author response:** *As for internal data validation, we envision different levels. Firstly, data analytical validation of results, internally but also comparing cross-cohort results takes place. We further have experimental validation, for example, the assessment of certain mixtures not only in populations, but also in vitro (cell models, organ models) and animal studies.

# Reviewer report: Reviewer response for version 1

**DOI:** 10.21956/openreseurope.20658.r49291

**Published:** 2025-02-19

**Authors:** Lyuba Varticovski

**Affiliations:** 1 National Institutes of Health, Maryland, USA

The ENDOMIX project addresses a critical gap in research regarding the impact of EDCs on human health, with a particular emphasis on the immune system—an area that has been insufficiently explored in prior studies. This work is essential for gaining a comprehensive understanding of the extent of EDCs' effects on all living organisms, including both human and animal health.

 The main weaknesses of the present manuscript lie in the details of the design and execution of these studies. For instance, the authors claim that “ENDOMIX will design in vitro studies to analyse endpoints relevant for immunotoxicants and associated mediators of health outcomes, as defined in the cohort studies.” These are two major undertakings that will require significant time and research investment.

 Firstly, there are no established in vitro methods for assessing the immune system effects of low-level EDCs typically found in the environment, which are usually present at nano and picomolar concentrations. The authors do not appear to consider the labour-intensive and costly techniques currently used in studies on mammals and other species. Existing methodologies should be adapted to study biologically active relevant EDC concentrations in living cells, such as tracking the nuclear translocation of activated hormonal receptors.  These are performed by analysing the nuclear/cytoplasmic ratio as the direct biological effect (PMID: 32018941). While such studies are pertinent to the immune system, assessing the nuclear/cytoplasmic ratio may present challenges when applied to immune cells.

 Further references to existing methodologies (PMID 34339825), along with the authors' commentary on their potential applications in immune system research, are necessary to support the continuation of the ENDOMIX studies.

 Secondly, to our knowledge, no established methodologies currently exist for linking *in vitro* studies to the associated mediators of health outcomes. All previous information has been based on correlations rather than direct evidence. If the authors intend to propose a novel approach for identifying the direct effect of EDCs on the immune system, it would be a significant advancement and would require a thorough, detailed explanation.

Where applicable, are recommendations and next steps explained clearly for others to follow? (Please consider whether others in the research community would be able to implement guidelines or recommendations and/or constructively engage in the debate)

Yes

Does the article adequately reference differing views and opinions?

No

Are all factual statements correct, and are statements and arguments made adequately supported by citations?

No

Is the rationale for the Open Letter provided in sufficient detail? (Please consider whether existing challenges in the field are outlined clearly and whether the purpose of the letter is explained)

Yes

Is the Open Letter written in accessible language? (Please consider whether all subject-specific terms, concepts and abbreviations are explained)

Yes

Reviewer Expertise:

Endocrine disruptors, Hormonal actions

I confirm that I have read this submission and believe that I have an appropriate level of expertise to confirm that it is of an acceptable scientific standard, however I have significant reservations, as outlined above.

## References

[1] : Endocrine disruptors of sex hormone activities. *Mol Cell Endocrinol* .2022;539: 10.1016/j.mce.2021.111415 111415 10.1016/j.mce.2021.111415 34339825 PMC8762672

[2] : Pilot study of global endocrine disrupting activity in Iowa public drinking water utilities using cell-based assays. *Sci Total Environ* .2020;714: 10.1016/j.scitotenv.2019.136317 136317 10.1016/j.scitotenv.2019.136317 32018941 PMC8459208

# Response

**Published:** 2025-11-14

**Authors:** Thi Anh Mai Pham

We appreciate the reviewers’ valuable comments and suggestions on our manuscript and would like to thank you for classifying the project as relevant and timely. We have carefully considered the points raised and prepared a detailed, point-by-point response to each comment, indicating the corresponding changes made in the manuscript and where they can be found in the revised version: 

**The main weaknesses of the present manuscript lie in the details of the design and execution of these studies. For instance, the authors claim that “ENDOMIX will design in vitro studies to analyse endpoints relevant for immunotoxicants and associated mediators of health outcomes, as defined in the cohort studies.” These are two major undertakings that will require significant time and research investment.** **Firstly, there are no established in vitro methods for assessing the immune system effects of low-level EDCs typically found in the environment, which are usually present at nano and picomolar concentrations. The authors do not appear to consider the labour-intensive and costly techniques currently used in studies on mammals and other species. Existing methodologies should be adapted to study biologically active relevant EDC concentrations in living cells, such as tracking the nuclear translocation of activated hormonal receptors.  These are performed by analysing the nuclear/cytoplasmic ratio as the direct biological effect (PMID: 32018941). While such studies are pertinent to the immune system, assessing the nuclear/cytoplasmic ratio may present challenges when applied to immune cells. Further references to existing methodologies (PMID 34339825), along with the authors' commentary on their potential applications in immune system research, are necessary to support the continuation of the ENDOMIX studies.**

***Author response:*** Thank you for this valuable comment. We acknowledge that the methodological options for assessing the impact of chemicals at real-life concentrations remain limited, and addressing this gap is precisely where the ENDOMIX project aims to make a difference. It is important to distinguish between screening methods that can evaluate multiple chemicals based on general endpoints with regulatory potential, and more sophisticated methods that provide a detailed mechanistic understanding of how chemicals affect the immune system.

 In ENDOMIX, we employ a tiered approach to investigate the effects of chemicals and their mixtures across models of increasing physiological complexity, ranging from cell-based systems to in vivo models. The first tier comprises high-throughput assays designed to measure molecular initiating events and key biological responses, such as the quantitative activation of nuclear receptors and transcription factors in reporter gene assays (e.g., estrogen receptor, NF-κB, thyroid receptors).

 For the high-throughput systems, we test mixtures at concentration ratios corresponding to those detected in environmental samples, but at slightly higher absolute concentrations to ensure measurable effects. Provided that these effects fall within the linear range of the concentration–response curves and linearity at low doses has been established, it is then possible to extrapolate the results to environmentally relevant concentrations.

 Regarding the papers mentioned by the reviewer, we would like to note that the high-throughput in vitro assays used in ENDOMIX follow similar principles to those described in the first cited paper, which in fact references our earlier work on the application of reporter gene assays for water quality assessment. The main difference is that their setup is not high-throughput, as it relies on 96-well plates. The review article cited is indeed an excellent summary of endocrine disruptors acting on sex hormone pathways and describes many of the assays typically used in this field. We have extensive experience with these types of assays and have carefully considered them in our design. However, since our focus in ENDOMIX is on immunomodulatory endpoints, which are novel in this context, we prioritized selecting and optimizing assays relevant to immune function.

 The second tier involves more advanced methods that go beyond single endpoints by employing primary immune cells (PBMCs) obtained from donor blood samples. We have already established flow cytometry protocols to assess the impact of chemicals on multiple immune cell subsets (doi: 10.3389/fimmu.2023.1327960). Within ENDOMIX, we will further refine these methodologies to characterize how chemical mixtures influence immune cell subsets, functional parameters such as cytokine secretion, and activation status. This approach also allows us to identify immune cell subpopulations warranting more detailed investigation. Subsequently, we are developing specific assays for selected immune cell types to gain a deeper mechanistic understanding of chemical effects at the single-cell level.

 For clarification, we have added a new paragraph under the section “ENDOMIX conception” providing the explanation mentioned above.

**Secondly, to our knowledge, no established methodologies currently exist for linking in vitro studies to the associated mediators of health outcomes. All previous information has been based on correlations rather than direct evidence. If the authors intend to propose a novel approach for identifying the direct effect of EDCs on the immune system, it would be a significant advancement and would require a thorough, detailed explanation.**

***Author response:*** We fully agree that establishing direct links between in vitro findings and health outcomes is inherently challenging. To address this complexity, ENDOMIX employs an innovative triangulation strategy that integrates evidence from cohort studies, in vitro, and in vivo models focusing on the same chemical mixtures. This multi-layered approach is designed to move beyond associative findings and towards the identification of causal immune targets of endocrine-disrupting chemicals (EDCs).

 In the cohort studies, chemical exposure data are linked to OLINK-derived proteomic profiles that capture subtle changes in immune parameters. This enables us to determine whether specific mixtures are associated with inflammatory signatures, immune suppression, or intracellular signaling alterations, and how these relate to defined health outcomes. By combining exposure data with molecular and clinical endpoints, we can generate biologically meaningful hypotheses that guide mechanistic testing.

 In the in vitro component, the same mixtures are systematically tested in human immune cell models to dissect their effects on inflammatory pathways, cytokine secretion, and immune functionality across defined cell subtypes. These mechanistic data provide insight into the cellular and molecular processes underlying the associations observed in human populations.

 In parallel, the in vivo studies allow us to assess whether the same mixtures can elicit or exacerbate specific health outcomes under controlled conditions, while concurrently evaluating their immunomodulatory effects. This offers a critical bridge between molecular mechanisms and organism-level responses.

 By integrating and cross-validating findings across these complementary tiers, ENDOMIX will generate a coherent, causality-oriented framework that connects environmental exposure to immune dysregulation and disease risk. This triangulated design represents a key strength of the project, enhancing both the robustness and translational relevance of the results.

 To address the reviewers’ comment, we have revised the paragraph describing the triangulated design under the section “ENDOMIX conception” according to the explanation mentioned above.
